# Nuclear genome assembly of *Leucinodes orbonalis* (Lepidoptera: Crambidae) collected from the Philippines

**DOI:** 10.1093/jisesa/ieaf066

**Published:** 2025-06-28

**Authors:** Joshua B Despabiladeras, Jimuel Adrian M Punzalan, Ma Anita M Bautista

**Affiliations:** Functional Genomics Laboratory, National Institute of Molecular Biology and Biotechnology, College of Science, University of the Philippines, Diliman, Quezon City, Metro Manila, Philippines; Functional Genomics Laboratory, National Institute of Molecular Biology and Biotechnology, College of Science, University of the Philippines, Diliman, Quezon City, Metro Manila, Philippines; Functional Genomics Laboratory, National Institute of Molecular Biology and Biotechnology, College of Science, University of the Philippines, Diliman, Quezon City, Metro Manila, Philippines

**Keywords:** whole genome sequencing, whole genome assembly, gene annotation, pest genomics

## Abstract

The eggplant fruit and shoot borer (*Leucinodes orbonalis* Guenée) is a devastating lepidopteran pest of the eggplant (*Solanum melongena* L.), causing significant economic losses. Reference genomes aid in understanding insect pest biology and can guide pest management programs. For eggplant fruit and shoot borer, however, genomic resources are scarce; hence, this study presents an annotated genome assembly of the Philippine eggplant fruit and shoot borer genome using Illumina short reads. The 480,399,388 bp long assembly contained 31,568 contigs with an N50 of 204,698 bp and a BUSCO score of 96.5%. Annotation of repeat elements indicates that the eggplant fruit and shoot borer genome comprises 38.50% interspersed repeats, which are mostly unclassified repeats. Functional RNA annotation revealed 1,310 functional RNA genes consisting primarily of tRNAs, rRNAs, snRNAs, and miRNAs. Protein structural annotation predicted a total of 12,671 genes. Annotation using a Cluster of Orthologous groups indicates proteins belonging to group S (unknown), group T (signal transduction), group O (posttranslational modification), and group K (transcription). Of the proteins belonging to group S, PFAM analysis revealed proteins containing chitin, juvenile hormone, odorant and pheromone-binding protein domains, and zinc finger motifs. Further analysis of the predicted proteins indicates that the EFSB possesses conserved biochemical machineries in insect chemosensation, detoxification, and hormone biosynthesis and reception. Variation profiling, on the other hand, detected 11,103,848 SNPs and 3,031,155 indels possibly occurring in Philippine eggplant fruit and shoot borer. Overall, the genome assembly and annotation generated from this study contribute to establishing genome references, and may aid in understanding the EFSB through future studies aimed at its effective control.

## Introduction

The solanaceous crop, *Solanum melongena* L., commonly known as the eggplant, aubergine, or brinjal, is one of the most important global crops (Gürbüz et al. 2018). In the Philippines, the eggplant accounts for 30% of the country’s total crop yield, providing a ready-source income for farmers (Hautea et al. 2014, 2016). However, chronic pest infestations hamper the consistent production of the eggplant. The eggplant fruit and shoot borer (EFSB), *Leucinodes orbonalis* Guenée, is one of the more serious pests of the eggplant, *S. melongena* L., causing widespread devastation in eggplant-producing countries of South and Southeast Asia (Navasero et al. 2016). The larval stage of the pest bores into the eggplant during its life cycle, causing tissue destruction and necrosis (Mainali 2014). The damage causes the eggplants to be unfit for marketing, with yield losses up to 80% (Hautea et al. 2016, Patel et al. 2018, Shelton et al. 2018).

Several different strategies have been employed to manage EFSB infestations such as insecticide treatments, resistant cultivar development, sex pheromone traps, and biological control (Ramasamy 2008, Gautam et al. 2019) with varying degrees of success. In eggplant-producing countries, insecticide spraying remains the widespread method of EFSB control, but the continual application of broad-spectrum insecticides causes significant health and environmental risks, and resistance development (Shelton 2010, Del Prado-Lu 2015, Shirale et al. 2017, Shelton et al. 2018). Recent advancements in the integration of the *Bacillus thuringiensis* Cry toxins into eggplants show promise in controlling EFSB infestations without the drawbacks of insecticide applications (Shelton 2010, Hautea et al. 2016, Navasero et al. 2016). However, widespread adoption of the technology has faced regulatory and cultural concerns (Shelton 2010, Hautea et al. 2016). Further understanding of EFSB biology can aid the development of complementary control strategies that can be implemented alongside current control strategies, including the use of *Bt* eggplant.

Hormonal control of developmental processes is important in the physiology of holometabolous insects like EFSB, as such make an attractive target for pest management. Proof-of-concept knockdown studies of key enzymes involved in hormone biosynthesis showed larval mortality in *Maduca sexta* (Yin et al. 2020), inhibited oviposition in *Helicoverpa armigera* (Wang et al. 2013), and growth retardation in *Chilo suppressalis* (Zhu et al. 2016). Similarly, insect detoxification enzymes are biochemical defenses insects employ to protect themselves from xenobiotic compounds. Knocking down detoxification enzymes or proteins involved in their transcriptional control have been shown to enhance susceptibility of the pests to various insecticides (Bai-Zhong et al. 2020, Peng et al. 2022, Fan et al. 2023, Yang et al. 2024, Hu et al. 2025). Thus, identifying the proteins involved in hormone biosynthesis and insect detoxification in EFSB may aid in the development of pest management schemes for this agriculturally important pest.

A suitable reference genome is paramount to a thorough understanding of organisms. The advancement of next-generation sequencing has led to significant development in the establishment of reference genomes, such as those involved in the i5K initiative (i5K Consortium 2013) and the Earth BioGenome Project (Lewin et al. 2018). However, genomic references for the EFSB are scarce, with 1 nuclear genome, ASM1942567v1, and 1 mitochondrial genome, PP493058 (Despabiladeras and Bautista 2024). The deposited nuclear genome of the EFSB came from EFSB populations in India, but mitochondrial COI analysis indicates differences in EFSB populations originating from different countries (Chang et al. 2016). Thus, it is important to obtain a reference genome for the Philippine EFSB population as part of the development of control strategies that complement current methods, including the adoption of *Bt* eggplant in the country. In this study, we present a whole genome assembly of the Philippine EFSB genome using short Illumina sequencing reads. The nuclear genome assembly presented here can serve as genetic resources for the EFSB.

## Materials and Methods

### Sample Collection, Nucleic Acid Extraction, and Library Preparation

Market-rejected eggplants obtained from Danso, Gerona, Tarlac; Santiago, Gerona, Tarlac; Laoag, Ilocos Norte; Batac, Ilocos Norte and Ilagan, Isabela were individually dissected and probed for EFSB infestations. The Institute of Plant Breeding (IPB) at the College of Agriculture and Food Science, University of the Philippines Los Baños also provided samples they collected and reared. For genomic DNA extraction, EFSB samples were stored at −80 °C and whole individuals were used for all 8 libraries. Multiple libraries of the same insert size were constructed to increase sequence coverage of the final assembly. The genomic DNA extraction, library preparation, and sequencing followed the methodology described in Despabiladeras and Bautista (2024). The samples utilized for DNA extraction and relevant metadata have been registered under BioProject PRJNA1211110, and BioSamples SAMN46265538 to SAMN46265545 while the raw genomic sequences have been deposited to the Sequencing Read Archive (SRA) with the following accession numbers: SRR32012941 to SRR32012948.

For RNA extraction, individuals of EFSB were stored in tubes containing TRIzol reagent (Ambion, USA) at −80 °C. Individual EFSB larvae were homogenized in 1 ml TRIzol reagent using a mortar and pestle. The homogenate was then transferred to sterile 1.5 ml tubes and incubated at room temperature for 5 min. Two hundred microliter of chloroform was then mixed with the homogenate and incubated for 3 min at room temperature. Next, the homogenate–chloroform mixture was then centrifuged at 12,000 × *g* for 15 min at 4 °C. The aqueous phase was carefully transferred to a new, sterile 1.5 ml tube, mixed with 500 µl of isopropanol, and incubated at room temperature for 10 min. A second centrifugation step was done with the same conditions described previously. The obtained RNA pellet was then washed with 75% ethanol, and re-pelleted using 7,500 × *g* for 5 min at 4 °C. The RNA extracts were then re-suspended in 30 µl of nuclease-free water for downstream analyses. To remove possibly contaminating DNA, DNAse treatment was then performed on the extracts using the TURBO DNA-free kit (Ambion, USA) following the manufacturer’s protocol. A second round of purification was done using the RNA Clean & Concentrator kit (Zymo Research, USA) to increase the purity for reverse transcription and sequencing. The RNA extracts were assessed using the Qubit RNA BR Assay (Thermo Fisher, USA), and by running on a 1% agarose gel in Tris–Borate–EDTA (TBE) buffer at 90 V for 45 min visualized with GelRed (Biotium Inc., Fremont, CA, USA) staining.

Library preparation from the purified RNA samples was performed using the TruSeq Stranded Total RNA (H/M/R) Library preparation kit (Illumina, USA). The quality and quantity of the cDNA libraries were checked via Qubit dsDNA BR Assay (Thermo Fisher, USA) and TapeStation (Agilent, USA) analysis. Paired-end cDNA sequencing was then done using the Illumina NovaSeq 6000 using a 2 × 150 bp run for 300 cycles at the Philippine Genome Center. The samples utilized for RNA extraction and relevant metadata have been registered under BioProject PRJNA768936 and the raw RNA-sequencing reads have also been deposited to the SRA with the following accession numbers: SRR16214354 to SRR16214382.

### Read Preprocessing, Read Filtering, and Genome Assembly

Raw sequences were preprocessed using fastp (Chen 2023) to remove adapter and polyG sequences and assessed using FastQC (Andrews 2023). Contaminating mitochondrial genome (mitogenome) sequences were removed by mapping the processed sequences to reference mitogenome sequences using Bowtie2 (Langmead and Salzberg 2012). Mitogenome references were obtained from NCBI organelle genome database and chosen based on the following criteria: close phylogenetic relationship with EFSB, tagged as a complete reference by NCBI, and must be reported or part of a published material. Based on these criteria, the following mitochondrial genomes were chosen: the *Glyphodes quadrimaculalis* mitogenome, NC_022699.1 (Park et al. 2015); *Maruca testulalis* mitogenome, NC_024283.1 (Zou et al. 2016); and *Omiodes indicata* mitogenome, NC_039177.1 (Yang et al. 2018). After aligning to the reference mitogenomes, the unmapped sequences were mapped to the reference PhiX genome (Sanger et al. 1977), NC_001422.1, to further clean the sequences. The genome size of the EFSB was estimated by counting *k*-mers of length 21 and 31 using Jellyfish (Marçais and Kingsford 2011), and then using Genomescope 2.0 (Ranallo-Benavidez et al. 2020) to estimate the genome size. The unmapped sequences were then used as the starting input for assembly using MaSuRCA (Zimin et al. 2013). Each of the 8 libraries were assembled separately, and then merged using ntjoin (Coombe et al. 2020) to obtain a combined draft assembly of the EFSB. The combined assembly was gap-filled using Ntedit and Sealer (Li et al. 2022). A final refinement was done by aligning EFSB RNA-sequencing reads with STAR (Dobin et al. 2013) to the combined assembly and using the alignment files to combine contigs through Rascaf (Song et al. 2016). The quality of the assembly was assessed using QUAST (Mikheenko et al. 2018) and BUSCO (Manni et al. 2021). Internal consistency was assessed by aligning the input reads to the assembly using BWA-MEM (Li 2013), and merging the resulting BAM files using samtools (Danecek et al. 2021). Coverage analysis was done using mosdepth (Pedersen and Quinlan 2018), and the insert size distribution was determined using picard tools (Broad Institute 2019). The merged assembly for the EFSB was deposited in NCBI with accession number JBLEQA000000000.

### Repeat Masking and Genome Annotation

After obtaining the refined and combined assembly, repeats were annotated and masked using RepeatModeler (Flynn et al. 2020) and RepeatMasker (Smit et al. 2013). In brief, a repeat database was built using the combined EFSB assembly and was then used by RepeatModeler to annotate repeats. RepeatMasker then used the annotated repeat families to mask the combined assembly. Annotation of the EFSB RNA genes was done using tRNAScanSE (Chan et al. 2021) to annotate tRNAs, and using Infernal (Nawrocki and Eddy 2013) coupled with RFAM 14 (Kalvari et al. 2021) to annotate other functional RNAs. Structural annotation of protein-coding gene models was then done using BRAKER3 (Hoff et al. 2019, Gabriel et al. 2021, 2023), combining AUGUSTUS ab initio prediction, arthropod protein homologs (Brůna et al. 2021), and extrinsic evidences from RNA-sequencing data (Hoff et al. 2016, Brůna et al. 2023). Prepartitioned arthropod protein homologs were obtained from OrthoDB (Kuznetsov et al. 2023). The RNA-sequencing reads were aligned to the combined assembly using STAR (Dobin et al. 2013). Both the arthropod orthoDB protein homologs and the RNA-sequencing BAM files were then provided to BRAKER3 for structural annotation. The completeness and consistency of the predicted EFSB proteome were then assessed using BUSCO (Manni et al. 2021) and Omark (Nevers et al. 2024). Functional annotation of the EFSB proteome was done using the eggnog-mapper tool (Cantalapiedra et al. 2021) with a Diamond (Buchfink et al. 2015) sensitivity of ultra-sensitive and Diamond iterative searches turned on, and Interproscan (Jones et al. 2014) using version 5.69-101.0 with the following options on: iprlookup, pathways, goterms. SignalP 4.1 (Nielsen 2017) and TMHMM (Krogh et al. 2001) were also activated during Interproscan annotation. Analysis of important gene families such as those involved development, hormone signaling and detoxification was done using the Preferred_name, EC and PFAMs fields in the eggnog-mapper output. A summary of all the important genes and gene families, PFAM domains and other information are given in the Supplementary Information.

### Analysis of Genetic Variations in the EFSB

Genetic variations occurring in the EFSB were determined by single nucleotide polymorphism (SNP) and insertion–deletion (Indel) profiling using the Genome Analysis Toolkit (GATK) (Auwera and O’Connor 2020). The individual sequencing reads were aligned to the merged assembly using BWA-MEM (Li 2013). The resulting BAM files were then appended with read groups and subsequently deduplicated using GATK AddOrReplaceReadGroups and MarkDuplicates, respectively. Initial variant calling was then done using GATK HaplotypeCaller. The resulting variant calls were merged using bcftools merge (Danecek et al. 2021) to obtain a collated set of variants, and then separated according to variant type using GATK SelectVariants. Variants were then filtered using the GATK recommendations for hard-filtering (Van Der Auwera et al. 2013) using VariantFiltration and SelectVariants. For SNPs, the following filters were applied: Quality by depth (QD) < 2.0, Quality (Qual) < 30, FisherStrand (FS) > 55.0, RMS Mapping Quality (MQ) < 40.0, Strand Odds Ratio (SOR) > 3.0, Mapping Quality Rank Sum Test (MQRankSum) < −12.5, and Read Pos Rank Sum Test (ReadPosRankSum) < −8.0. Similarly, for Indels, the following filters were applied: QD < 2.0, Qual < 30, FS > 200.0, and ReadPosRankSum < -20.0. After filtering the first round of variant calls, base quality score recalibration (BQSR) was done to recalibrate quality scores that may have been affected by systematic errors. After recalibration, a second round of variant calling and filtering was done using the recalibrated BAM files to obtain filtered high-quality variants. The variant calling statistics were then summarized using bcftools (Danecek et al. 2021).

## Results and Discussion

### EFSB Draft Genome Assembly

A total of 1,057,066,000 high-quality sequencing reads were obtained after fastp filtering and removal of mitochondrial sequences. K-mer-based estimation of the EFSB genome size using GenomeScope 2.0 (Ranallo-Benavidez et al. 2020) indicated that the genome size is estimated to be 455 to 469 Mb (Supplementary Fig. S1). The size is close to the flow cytometry result of Gandhi Gracy et al. (2019) where the haploid genome size of EFSB was found to be *C* = 472.26 Mb (2*C* = 944.52 Mb). A summary of the quality statistics for each library post-filtering is given in Supplementary Fig. S2. Each post-filtering step outlined slight improvements in assembly quality, as indicated by the marginal increase in BUSCO score using the lepidoptera_odb10 dataset (Supplementary Fig. S3). The final combined assembly has a total length of 480,339,388 bp, N50 score of 204,698 bp and a BUSCO score of 96.5% on the lepidoptera_odb10 dataset (Table 1). The sequencing reads were aligned to the combined assembly to determine the sequencing coverage and as a final check of quality. A total of 1,042,350,195 reads were mapped to the merged assembly, constituting 98.61% of the total reads, with 87.39% of the total reads being properly paired, indicating good internal consistency. Coverage analysis using mosdepth revealed that 96% and 91% of the total genome was covered at least 30× and 100×, respectively, indicating good sequencing depth (Supplementary Fig. S4A). Insert size estimation showed that the distribution of the insert size has a mean length of 500 bp (Supplementary Fig. S4B), which is close to the expected insert size from the library preparation. Overall, based on the QUAST and BUSCO metrics, and internal consistency, the obtained merged assembly of the EFSB is of good quality and is suitable for downstream analyses.

**Table 1.** Statistics for the draft genome assembly and annotation of *Leucinodes orbonalis*, EFSB

Assembly statistics	
Number of contigs	31,568
Largest contig (bp)	1,739,805
Total Assembly Length (bp)	480,339,388
N50 (bp)	204,698
GC content	36.56%
BUSCO score	96.5%
Annotation statistics	
Number of predicted genes	12,671
Total predicted mRNAs	16,065
Mean gene length (bp)	9,457
Mean mRNA length (bp)	11,311
Total number of exons	106,408
Total number of introns	90,343
Mean exon length (bp)	217
Mean intron length (bp)	1756
Mean exons per mRNA	6.6
Mean introns per mRNA	5.6
Mean mRNAs per gene	1.3

### Annotation of RNA Genes

**Fig. 1.**
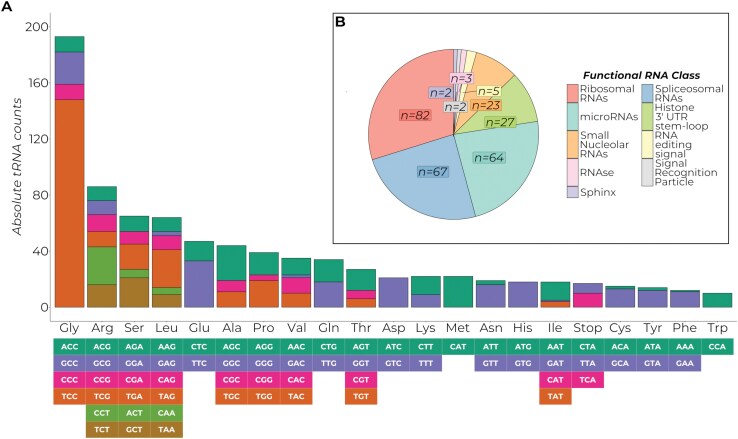
Summary of the annotated RNA genes in the genome of *Leucinodes orbonalis*, EFSB. A) Distribution of annotated tRNAs according to amino acid isotype and codon sequence. The colors show the distribution of tRNA codons for each amino acid indicated by the legend below the plot. B) Pie chart showing the non-tRNA functional RNA genes determined by Infernal. The general classification of the annotated RNA genes is indicated in the legend and shown in decreasing order of abundance.

A total of 1,035 putative tRNA genes in the EFSB genome were annotated, with 439 (42.42%) genes decoding for the standard 20 amino acid tRNAs and 532 (51.40%) putative tRNA pseudogenes. The remaining tRNA genes were mismatched or have undetermined isotypes. Out of the 439 functional tRNA genes, 79 contained introns. A particular abundance of glycine tRNAs was found, mostly due to the TCC codon (Fig. 1A), followed by arginine, serine, and leucine. No particular pattern or association of codon usage with GC content can be observed.

For the remaining functional RNA genes, a total of 275 RNA genes were annotated by Infernal (Fig. 1B). The majority consist of ribosomal RNAs (rRNA), with 82 rRNA genes annotated. The rRNA genes consist of important RNA elements necessary for the functioning of the ribosome, such as the 28S rRNA, 5.8S rRNA, 5S rRNA of the large ribosomal subunit, and the 18S rRNA of the small ribosomal subunit (Baßler and Hurt 2019). The next major RNA class is the spliceosomal small nuclear RNAs (snRNAs) with the EFSB having a total of 67 such genes. The annotated snRNAs consist of the 5 core RNA genes important for the functioning of the major spliceosome (U1, U2, U4, U5, and U6) and the 4 atypical snRNA genes of the minor spliceosome (U11, U12, U4atac, and U6atac) (Karijolich and Yu 2010). Infernal also annotated a diverse class of microRNAs (miRNAs) from a wide range of miRNA families in the EFSB genome, with 64 genes. Other important functional RNAs such as the small nucleolar RNAs important for proper rRNA biogenesis (Wu et al. 2019), and the conserved signal recognition particle important for translocation into the endoplasmic reticulum (Akopian et al. 2013) were also annotated in the EFSB genome (Fig. 1B).

### Annotation of Repetitive Elements in the EFSB Genome

After obtaining a working assembly for the EFSB genome, the repetitive elements of the EFSB genome were annotated and masked for subsequent protein prediction and annotation. RepeatModeler annotated a total of 184,945,958 bp of interspersed repeats, constituting 38.50% of the assembly length. The majority of the annotated repeats were unclassified repeats that constitute 28.33% of the genome (Table 2). The long interspersed nuclear elements (LINEs) and DNA transposons were also detected in the EFSB genome, with TcMar-Mariner and RTE constituting the majority of LINEs and DNA transposons, respectively (Table 2).

**Table 2.** Repeat families annotated by RepeatModeler in the genome of *Leucinodes orbonalis*, EFSB

Repeat type/family	Number of elements	Total length occupied (bp)	Percentage (%)
**DNA**	173	47,022	0.01
CMC-Chapaev-3	563	197,823	0.04
CMC-Transib	1,773	104,879	0.02
Ginger-2	66	10,168	0.00
Maverick	30	8,846	0.00
P	671	260,182	0.05
PIF-Harbinger	48	15,642	0.00
PIF-ISL2EU	38	10,499	0.00
PIF-Spy	23	4,992	0.00
PiggyBac	202	118,216	0.02
Sola-1	98	47,125	0.01
Sola-2	195	104,511	0.02
TcMar-Fot1	117	61,398	0.01
TcMar-Mariner	7,841	1,007,218	0.21
TcMar-Tc1	489	155,414	0.03
TcMar-Tigger	258	82,317	0.02
TcMar-m44	97	37,524	0.01
hAT-Ac	405	113,480	0.02
hAT-Blackjack	222	69,797	0.01
hAT-Tip100	154	69,588	0.01
**LINE**	23,058	2,626,830	0.55
CR1	9,778	2,105,814	0.44
CR1-Zenon	28,866	4,921,694	1.02
CRE	5,203	1,003,958	0.21
Dong-R4	1,077	247,420	0.05
I	1,129	378,291	0.08
I-Jockey	20,979	3,009,897	0.63
L2	52,662	11,549,937	2.40
Proto2	3,504	687,602	0.14
R1	2,151	1,090,649	0.23
R1-LOA	4,118	940,875	0.20
RTE-BovB	5,797	1,167,446	0.24
RTE-RTE	75,889	14,735,438	3.07
**LTR**	…	…	…
Copia	457	140,771	0.03
DIRS	780	266,145	0.06
Gypsy	2,085	840,862	0.18
Pao	947	485,354	0.10
**RC**	…	…	…
Helitron	54	10,038	0.00
**Unknown**	95,4127	136,114,971	28.33
Simple repeat	1,680	95,325	0.02
**OTAL**	1,207,804	184,945,958	38.50

Interestingly, the short interspersed nuclear elements (SINEs) were not annotated by RepeatModeler in the EFSB genome. These elements may have been obscured in the unclassified annotations. Overall, a total of 38.50% of the EFSB genome consists of interspersed repeats that were successfully masked prior to gene prediction and annotation.

### Structural and Functional Annotation of EFSB Protein-Coding Genes

**Fig. 2.**
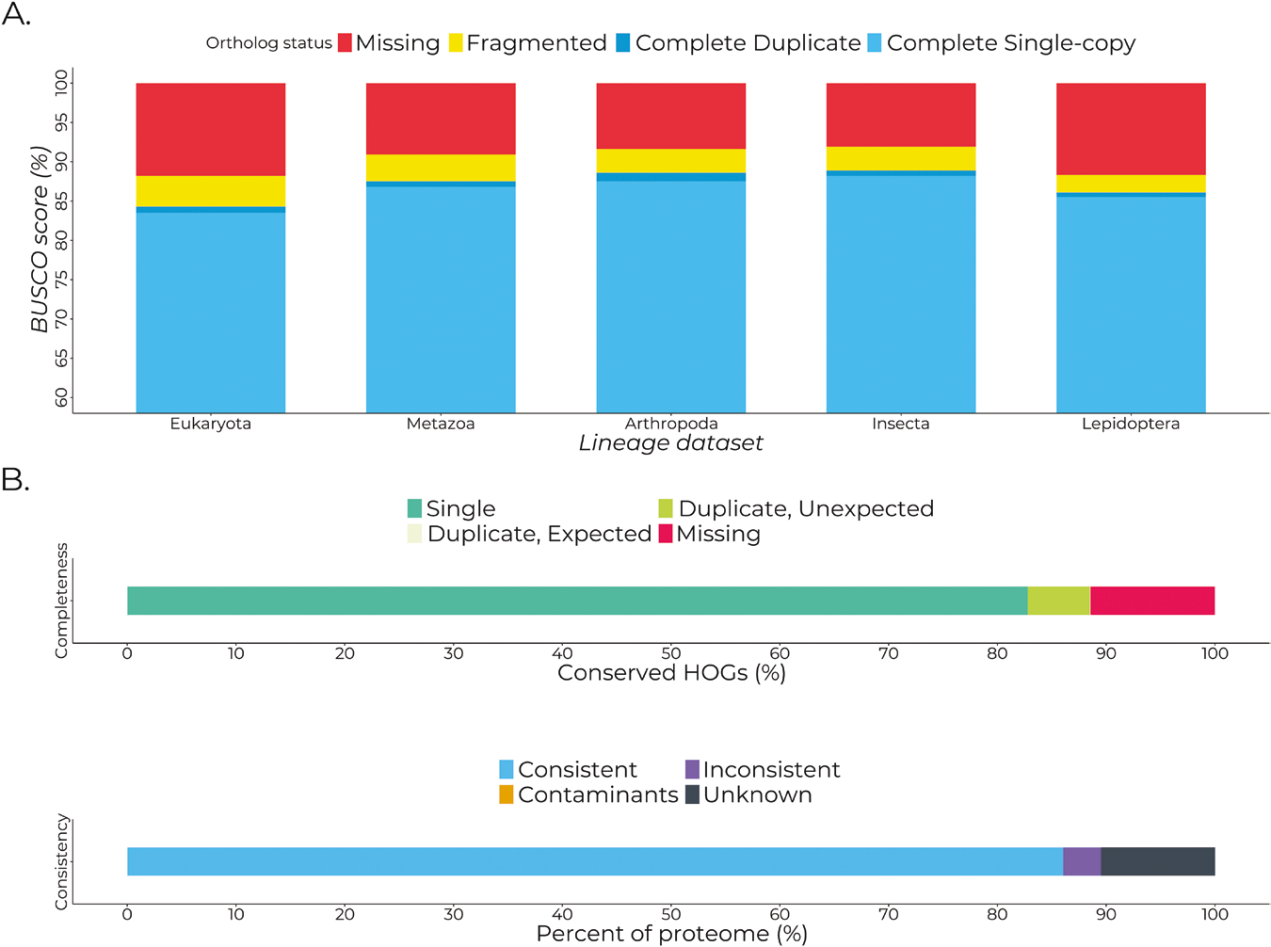
Completeness and consistency of the annotated proteome of *Leucinodes orbonalis*, EFSB assessed by BUSCO and Omark. A) BUSCO scores of the annotated EFSB proteome retaining only the longest isoforms assessed using different BUSCO lineages. All lineages used are the odb10 versions. B) Omark completeness and consistency checking of the EFSB proteome. Omark is similar to BUSCO but utilizes hierarchical orthologous groups (HOGs) for a specific clade instead of the universal orthologues utilized by BUSCO. Omark determined that the input proteome was from *Obtectomera*, consistent with the taxonomic placement of EFSB.

Structural annotation and prediction of open reading frames were done using BRAKER3 by combining ab initio prediction from AUGUSTUS, protein homologs from phylum *Arthropoda*, and extrinsic evidences from RNA-sequencing data. BRAKER3 annotated 12,671 genes spread across 16,065 transcripts, with a total of 106,408 and 90,343 exons and introns, respectively (Table 1). The EFSB has mean gene and transcript lengths of 9,457 and 11,311 bp, respectively. Introns are generally longer than the exons, with a mean length of 1,756 bp compared to 217 bp for exons. There are generally more exons per transcript in the EFSB compared to introns (Table 1). The higher number of transcripts than protein-coding genes indicates the presence of spliced isoforms of some genes that the RNA-sequencing data may have captured. BUSCO checking of the predicted EFSB proteome yielded an average 87.08% completeness across 5 BUSCO datasets, with 86.1% completion on the Lepidopteran dataset (Fig. 2A). Similarly, Omark checking yielded 88.55% completeness and determined that the EFSB proteome was 86.05% consistent with clade *Obtectomera*, agreeing with the supposed taxonomic placement of EFSB (Fig. 2B). Overall, the high completeness and consistency of the EFSB genome in the context of the designated clade indicate successful structural annotation with BRAKER3 (Fig. 2).

**Fig. 3.**
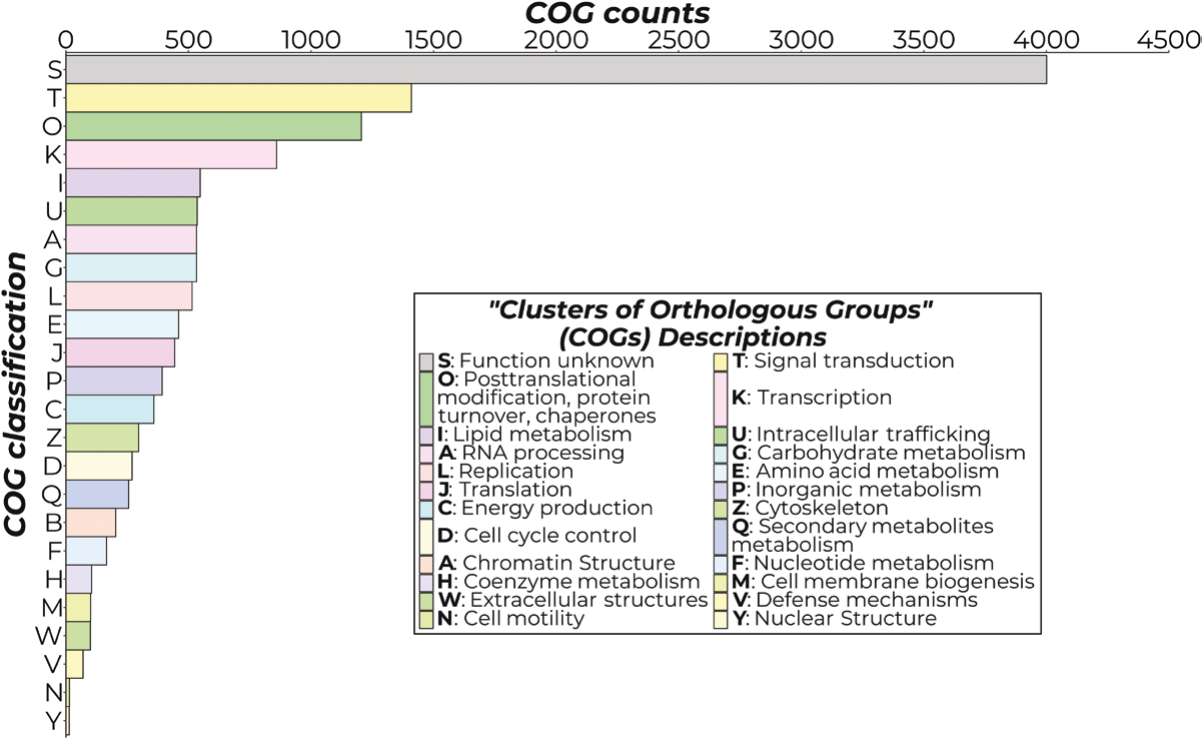
Grouping of the *Leucinodes orbonalis*, EFSB proteome based on COGs. The descriptions of the one-letter COG group abbreviation are given in the legend. The COGs are colored based on the hexadecimal color associated with each COG group as specified in the COG database.

After predicting putative gene models in the EFSB genome, functional annotation was done using eggnog-mapper to assign functions to the predicted genes based on orthologous groups and PFAM domains, respectively. Eggnog-mapper managed to assign and process 15,167 transcripts from the EFSB genome, representing 94.41% of the transcripts annotated by BRAKER3. Summarizing and grouping the processed hits according to Cluster of Orthologous Groups (COG) category indicates that 13,395 of the transcripts were successfully assigned to a COG group. A total of 4,001 transcripts were grouped in category S, which is the COG group having unknown functions (Table 3 and Fig. 3).

**Table 3.** Annotated protein-coding genes in the EFSB genome grouped according to COG categories

COG classification	COG description	Number of PCGs
A	RNA processing and modification	533
B	Chromatin structure and dynamics	203
C	Energy production and conversion	359
D	Cell cycle control, cell division, chromosome partitioning	270
E	Amino acid transport and metabolism	460
F	Nucleotide transport and metabolism	166
G	Carbohydrate transport and metabolism	533
H	Coenzyme transport and metabolism	105
I	Lipid transport and metabolism	548
J	Translation, ribosomal structure and biogenesis	444
K	Transcription	860
L	Replication, recombination and repair	515
M	Cell wall/membrane/envelope biogenesis	101
N	Cell motility	15
O	Posttranslational modification, protein turnover, chaperones	1206
P	Inorganic ion transport and metabolism	393
Q	Secondary metabolites biosynthesis, transport and catabolism	256
R	General function prediction only	0
S	Unknown function	4001
T	Signal transduction mechanisms	1410
U	Intracellular trafficking, secretion, and vesicular transport	536
V	Defense mechanisms	70
W	Extracellular structures	100
X	Mobilome: prophages, transposons	0
Y	Nuclear structure	14
Z	cytoskeleton	297
**TOTAL**		**13,395**

Based on COG classifications, the EFSB genome contains numerous proteins involved in signal transduction (Group T), posttranslational modification and protein turnover (Group O), and transcription (Group K). These results may relate to EFSB’s exposure to numerous biological and chemical challenges during its life cycle, and various signal transduction mechanisms are essential for the pest to respond to changing environments. Posttranslational modifications and transcription proteins are associated with this since the downstream targets for signal transductions are usually components involved in gene regulation.

Of the transcripts with a COG classification of “S: Unknown function”, a total of 2,447 transcripts has an associated PFAM domain annotation. Inspection of the PFAM domains show that 126 predicted transcripts have chitin binding activity (PFAM: Chitin_bind_4, PF00379). The PFAM domain PF00379 is associated with insect cuticle proteins having the R&R consensus domain believed to have chitin binding activity. The next most abundant PFAM domain in category S are the classical zinc finger transcription factors characterized by the conserved 2 cysteine and 2 histidine residues important for zinc coordination (PFAM: zf-C2H2, PF00096). Interestingly, category S also contains proteins having PFAM domains important for insect homeostasis such as juvenile hormone binding proteins or JHBPs (PFAM: JHBP, PF06585), pheromone-binding proteins (PBPs) (PFAM: OS-D, PF03392), and odorant-binding proteins (OBPs) (PFAM: PBP_GOBP, PF01395). JHBPs are insect-specific proteins that bind and protect Juvenile hormone from nonspecific hydrolysis in the hemolymph, and as such important proteins in insect metamorphosis and physiology (Li et al. 2016, Zhang et al. 2019b). PBPs and OBPs are important proteins involved in insect chemosensation. PBPs are small proteins that bind to pheromones, enhancing the specificity and sensitivity of the insect’s olfactory system to pheromones (Fang et al. 2018, Liu et al. 2023). Similarly, the OBPs are accessory proteins that bind to hydrophobic odorants, greatly enhancing solubility and binding to odorant receptors (Sun et al. 2018, Abendroth et al. 2023). Overall, a majority of the proteins annotated in the EFSB genome perform important housekeeping functions related to signal transduction, gene expression, and binding to important insect-related compounds.

### Insect Chemosensation and Detoxification

Insects encounter numerous stresses and challenges during their life cycle, and thus must be able to rapidly respond and adapt to survive. One of the key innovations in insect evolution is their adaptive and expansive chemoreception capabilities (Hansson and Stensmyr 2011, Agnihotri et al. 2016). The primary organ responsible for insect chemosensation is the sensillum that contains an aqueous fluid containing the pheromone binding and OBPs described previously (Hansson and Stensmyr 2011). Both protein families enhance the sensitivity of insect olfaction by solubilizing and transporting odorants to chemosensory receptors expressed in the chemosensory neurons of the sensillum (Vieira and Rozas 2011). Searching the EFSB annotations for both PBPs (PFAM: OS-D), and OBPs (PFAM: PBP_GOBP) show that the EFSB contains 22 and 18 PBPs and OBPs respectively (Table 4).

**Table 4.** Summary of the annotated *Leucinodes orbonalis*, EFSB chemosensation and detoxification genes

Protein superfamily	PFAM domain	PFAM description	PFAM number	No. annotated in EFSB
G-protein coupled receptors (GPCRs)	7tm_1	7 transmembrane receptor (Rhodopsin family)	PF00001	60
	7tm_2	7 transmembrane receptor (Secretin family)	PF00002	14
	7tm_3	7 transmembrane sweet-taste receptor of 3 GPCR	PF00003	5
Insect chemosensation				
Pheromone-binding proteins	OS-D	Insect pheromone-binding family, A10/OS-D	PF03392	22
Odorant-binding proteins	PBP_GOBP	PBP/GOBP family	PF01395	18
GRs and ORs	7tm_6	7tm Odorant receptor	PF02949	25
	7tm_7	7tm Chemosensory receptor	PF08395	5
Insect detoxification				
Cytochrome P450 monoxoygenases	P450	Cytochrome P450	PF00067	52
Carboxyl-esterasaes	COesterase	Carboxylesterase family	PF00135	74
	Abhydrolase_2	Phospholipase/ Carboxylesterase	PF02230	2
UDP	UDPGT	UDP-glucoronosyl and UDP-glucosyl transferase	PF00201	26
GST	GST_N	Glutathione S-transferase, N-terminal domain	PF02798	27
	GST_N_3		PF13417	
	GST_N_4		PF17172	
	GST_C	Glutathione S-transferase, C-terminal domain	PF00043	
	GST_C_2		PF13410	
	GST_C_3		PF14497	
	GST_C_6		PF17171	

ABC	ABC_membrane	ABC transporter transmembrane region	PF00664	42
	ABC_tran	ABC transporter	PF00005	
	ABC_tran_Xtn		PF12848	
	MTABC_N	Mitochondrial ABC-transporter N-terminal five TM region	PF16185	
	ABC_membrane_2	ABC transporter transmembrane region 2	PF06472	
	ABC_transp_aux	ABC-type uncharacterized transport system	PF09822	
	ABC2_membrane	ABC-2 type transporter	PF01061	
	ABC2_membrane_3	ABC-2 type transporter protein	PF12698	

The chemosensory receptors are primarily composed of the gustatory receptors (GRs) and the odorant receptors (ORs), which are transmembrane proteins characterized by 7 transmembrane α-helices (7TMs) (Sokolinskaya et al. 2020, Wicher and Miazzi 2021). The ORs are believed to have evolved from the more ancient GRs responsible for taste perception (Sokolinskaya et al. 2020), and are one of the adaptive novelties responsible for the evolutionary radiation of insects (Brand et al. 2018). The GRs and ORs are highly similar to G-protein coupled receptors (GPCRs) due to their conserved 7TMs (Wicher and Miazzi 2021). Searching the EFSB proteome for annotated PFAMs containing the 7TMs yielded a total 109 genes divided into 5 PFAM families (Table 4). The EFSB genome contains 60 genes belonging to the rhodopsin-like GPCRs (PFAM: 7tm_1, PF00001), 14 genes belonging to the secretin-like GPCRs (PFAM: 7tm_2, PF00002), and 5 genes belonging to the glutamate GPCRs (PFAM: 7tm_3, PF00003). The 3 families are part of the currently recognized 5 superfamilies of GPCRs (Krishnan et al. 2012) that have been shown to cluster distinctly from insect ORs and GRs (Nordstrom et al. 2011). Despite the presumed lack of role in chemosensation, the 3 GPCR families are involved in other important processes in insect physiology such as color perception, neuropeptide reception, metamorphosis, and immunity (Krishnan et al. 2012, Tran et al. 2019, Liénard et al. 2021).

The 7 transmembrane domains characteristic of insect GRs and ORs are divided into 2 PFAM families, namely the 7tm OR family (PFAM: 7tm_6, PF02949) and the 7tm chemosensory receptor family (PFAM: 7tm_7, PF08395) (Karpe et al. 2021, Bibi et al. 2023). The EFSB genome contains 25 genes belonging to the 7tm OR family, and 5 genes belonging to the 7tm chemosensory receptor family. Notably, 1 member of the 7tm_6 family was annotated to be the EFSB homologue of the OR co-receptor (Orco) gene. Odorant sensation in insects require the heterodimerization of the OR and the Orco gene (Sokolinskaya et al. 2020), with Orco being highly conserved in insects (Soffan et al. 2018). The annotated Orco of the EFSB is consistent with the Orco genes of both *Drosophila melanogaster* (UniProt accession: Q9VNB5) and *Anopheles gambiae* (UniProt accession: Q7QCC7) that contain 1 hit of the 7tm_6 PFAM domain.

Aside from chemosensation, another form of insect adaptation to stress, in particular xenobiotic stress, is biochemical detoxification. Detoxification reactions are commonly divided into 3 distinct phases: functionalization (Phase 1), conjugation (Phase 2), and excretion (Phase 3) (Kshatriya and Gershenzon 2024). Phase 1 detoxification generally involves oxidation and/or hydrolysis reactions that convert the xenobiotic to more polar derivatives, reducing toxicity and facilitating excretion. Phase 1 reactions are facilitated by the cytochrome P450 (p450s) and carboxylesterase (COEs) superfamilies (Nauen et al. 2022, Cruse et al. 2023). The P450s are diverse monooxygenases that utilize molecular oxygen to catalyze the transformation of distinct substrates such as pesticides, terpenes, phenolics, and alkaloids (Nelson et al. 2013, Nauen et al. 2022, Kshatriya and Gershenzon 2024). On the other hand, the COEs constitute an equally diverse superfamily containing the prototypical enzymes that catalyze hydrolytic reactions of distinct substrates (Yan et al. 2012, Wei et al. 2021), as well as noncatalytic esterases involved in neurodevelopmental functions (Durand et al. 2017, Cruse et al. 2023). The P450s are characterized by the PFAM domain, p450 (PF00067), while carboxylesterases can contain either the PFAM domains COesterase (PF00135) or Abhydrolase_2 (PF02230). Searching the annotations of proteins containing said domains indicate that the EFSB genome contains 52 P450s and 76 COEs (Table 4).

Phase 2 detoxification reactions involve the conjugation of R groups to xenobiotics or their Phase 1 intermediates to facilitate excretion, with the usual R groups being sugar or glutathione moieties catalyzed by UDP-glycosyltransferases (UGTs) and glutathione S-transferases (GSTs), respectively (Kshatriya and Gershenzon 2024). Both enzyme superfamilies are known to be involved in xenobiotic detoxification in different insect orders, highlighting the importance of these enzymes in biochemical adaptations of insects (Nagare et al. 2021, Koirala B K et al. 2022). The EFSB genome contains a total of 53 Phase 2 detoxification enzymes, with 27 GSTs and 26 UGTs (Table 4). Phase 3 reactions are governed by excretion mechanisms catalyzed primarily the ABC transporters in insects (Kshatriya and Gershenzon 2024). The ABC transporters are an evolutionary conserved superfamily characterized by the ATP-binding cassette (ABC) domain that serves to transport a multitude of substrates across the cell membrane (Heckel 2021). Indeed, insect ABC transporters have been implicated in the excretion of toxic xenobiotics such as insecticides (Dermauw and Van Leeuwen 2014). Checking the EFSB annotations for proteins containing different PFAM domains associated with ABC transporters shows that the EFSB genome contains 42 putative ABC transporter genes (Table 4). Lastly, the EFSB genome was also annotated to contain some members of conserved core machinery that underlies the transcriptional control of detoxification genes. For example, AhR/ARNT are basic helix-loop-helix (bHLH) and Per-ARNT-Sim (PAS) domain-containing transcription factors that are responsive to xenobiotic stress (Amezian et al. 2021). The EFSB genome was annotated to contain a single homologue of the ARNT gene that contains the bHLH (PFAM: HLH, PF00010) and PAS (PFAM: PAS, PF00989 and PFAM: PAS_11, PF14598) domains characteristic of the *D. melanogaster* homologue of ARNT (UniProt: O15945). Additionally, the EFSB genome also contains a single homologue of the double treble clef zinc finger domain-containing (PFAM: zf-C4, PF00105) nuclear hormone receptor 96 gene that has been shown to mediate xenobiotic responses in *D. melanogaster* (Afschar et al. 2016).

### Insect Hormone Biosynthesis and Signaling Components

The hormones, 20-hydroxyecdysone (20-E) and the juvenile hormone (JH) are 2 of the most important and ubiquitous hormone signals in insects. The EFSB draft assembly contains conserved core biosynthetic components of these hormones. The biosynthesis of the JH stems primarily from the universal mevalonate pathway that condenses acetyl-CoA to form the 5-carbon isoprenoid building blocks, isopentenyl pyrophosphate (IPP) and dimethylallyl pyrophosphate (DMAPP) (Supplementary Fig. S5A). The first step proceeds by the condensation of acetyl-CoA to acetoacetyl-CoA by the biosynthetic type 2 thioesterase. Notably, no direct annotation for a type 2 thioesterase was found in the EFSB genome. However, upon inspecting the enzyme commission (EC) annotation for biosynthetic type 2 thioesterases (EC2.3.1.9), there is one annotated gene that contains the characteristic thiolase N (PF00108) and C (PF02803) domains with an additional glutaredoxin-like domain (DUF836, PF05768). This gene may be the candidate biosynthetic type 2 thioesterase in the EFSB genome. After the formation of acetoacetyl-CoA, another molecule of acetyl-CoA is condensed by hydroxymethylglutaryl-CoA (HMG-CoA) synthase to form HMG-CoA, which is then reduced by HMG-CoA reductase to form mevalonate. Mevalonate then gets phosphorylated twice before undergoing a reductive decarboxylation step to form IPP. IPP and DMAPP are isomers and can be transformed into one another via IPP isomerase. Annotated enzymes important in the above steps, namely HMG-CoA synthase, HMG-CoA reductase, mevalonate kinase, and phosphomevalonate kinase, can be observed in the EFSB genome. The IPP and DMAPP are important isoprenoid building blocks for a variety of molecules such as hormones, dolichol, heme, and ubiquinone (Bellés et al. 2005). In particular, the enzyme farnesyl diphosphate synthase (FDPS) catalyzes the sequential addition of IPP and DMAPP to form farnesyl diphosphate (FPP), a notable key conserved intermediate branchpoint (Lombard and Moreira 2011). The EFSB genome contains multiple genes having an EC number of 2.5.1.10 corresponding to farnesyl diphosphate synthase activity. To synthesize JH, FPP gets dephosphorylated and then oxidized twice to form farnesoic acid (Noriega 2014). Notably, there is no direct annotation for the FPP phosphatase in the EFSB genome. There are 5 genes with PFAM domains of alkaline phosphatase (PFAM: Alk_phosphatase, PF00245). Searching for alkaline phosphatases associated with PWY-6650, the MetaCyc ID for JH-III biosynthesis yielded 5 hits, indicating that these genes may indeed be involved in the dephosphorylation of FPP during JH biosynthesis in EFSB. Upon formation of farnesol, it is oxidized twice via farnesol dehydrogenase and farnesal dehydrogenase to form farnesoic acid (FA). Two genes having the PFAM domain short chain dehydrogenase (PFAM: adh_short, PF00106) with EC 1.1.1.216 corresponding to farnesol dehydrogenase activity. Contrastingly, no annotated genes were in the EFSB genome with the EC 1.2.1.94, corresponding to farnesal dehydrogenase activity. However, searching for aldehyde dehydrogenases associated with PWY-6650 yielded 9 putative genes with such activity, indicating that at least one of these hits may be responsible for the conversion of farnesal to FA. In Lepidoptera, FA is oxidized before being methylated to form JH by farnesoate epoxidase and JH methyltransferase, respectively (Defelipe et al. 2011, Guo et al. 2021). Similarly, with farnesal dehydrogenase, there are no annotated genes in the EFSB genome with EC 1.14.13.203 or EC 1.14.14.128 corresponding to farnesoate epoxidase activity. However, narrowing down the Interpro annotations having both PWY-6650 and IPR002401 (Cytochrome P450, E-class, group I), yielded 43 hits that may be involved in the epoxidation of farnesoate. For the methyltransferase activity, there is a single annotated JH methyltransferase in the EFSB genome with an EC 2.1.1.325 corresponding to JH-III synthase activity.

An important metabolic feature of insects is their incapability of synthesizing sterol compounds, as they have lost the enzymes such as squalene synthase and sterol 14-demethylase (CYP51) necessary for converting FPP to squalene and zymosterol, key intermediates in sterol biosynthesis (Nelson et al. 2013, Zhang et al. 2019a). The hormone 20-E is a steroid hormone that is not biosynthesized using the classical mevalonate and sterol pathways, as in other metazoans (Niwa and Niwa 2014). As such, insects must obtain sterol precursors in their diet to enable ecdysteroid biosynthesis. In the case of EFSB, the eggplant, *S. melongena*, is a member of the *Solanaceae* which produces bioactive steroidal glycoalkaloids. Thus, *Solanaceae* members accumulate cholesterol as a major biosynthetic sterol, indicating that the EFSB can possibly obtain the necessary cholesterol from their eggplant diet (Sonawane et al. 2016, Barchi et al. 2019). The biosynthesis of ecdysteroids starts from the dehydration of cholesterol to form 7-dehydrocholesterol catalyzed by the Rieske-type oxygenase *neverland* (Niwa and Niwa 2014) (Supplementary Fig. S5B). The EFSB genome contains an annotated gene for the *neverland* gene and contains the PFAM domain Rieske (PF00355) with an EC number of 1.14.19.21 corresponding to cholesterol 7-desaturase activity characteristic of the *neverland* gene. The complete biosynthetic route of ecdysteroids contains gaps in the conversion of the 7-dehydrocholesterol to the 5β-ketodiol. termed as the Black Box (Niwa and Niwa 2014). The hypothetical metabolic steps occurring in the Black Box involve the oxidation of the 3β alcohol, formation of the 6-keto group, and hydroxylation of C14 to form a Δ^4^-diketol. The *spook* (spo) gene is thought to act in the Black box by catalyzing the conversion of 7-dehydrocholesterol to Δ^4^-diketol (Pondeville et al. 2013). The EFSB genome is found to have an annotated cytochrome P450 protein, CYP307A1, which is the putative candidate for the EFSB spo gene. Afterwards, the Δ^4^-diketol undergoes 2 successive reductions to form the 5β-ketodiol and is thought to be catalyzed by the *shroud* (sro) gene (Pondeville et al. 2013). The *D. melanogaster* sro gene is a short chain dehydrogenase (Niwa et al. 2010) (PFAM: adh_short, PF00106) with an enzyme annotation of EC 1.1.1.239 and 1.1.1.62. The EFSB genome contains a single gene with the same PFAM and EC annotations, making it the putative candidate for the EFSB sro gene. The 5β-ketodiol then undergoes 4 terminal hydroxylation steps catalyzed by the cytochrome P450 Halloween genes *phantom* (phm), *disembodied* (dib), *shadow* (sad) and *shade* (shd), respectively to form 20-E (Pondeville et al. 2013). The EFSB genome was found to have the cytochrome proteins CYP306A1, CYP302A1, CYP315A1, and CYP314A1 which are the putative candidates for the EFSB phm, dib, sad, and shd genes, respectively.

Hormone signaling in insects proceeds primarily through nuclear hormone receptors which are ligand-dependent transcription factors that induce expression in the presence of hormone ligands (Fahrbach et al. 2012, Cheatle Jarvela and Pick 2017). The receptor for JH in insects is the Methoprene-tolerant (Met) gene, a nuclear receptor containing both a bHLH and PAS domains (Charles et al. 2011, Ma et al. 2018). The EFSB contains a single homologue having the bHLH (PFAM: HLH, PF00010) and PAS (PFAM: PAS_11, PF14598) domains characteristic of the Met receptor. The EFSB genome also contains a homologue of the downstream effector of JH signaling, the Krüppel homolog 1 (Kr-h1) which represses the expression of ecdysone-response genes such as the Broad-complex, Br-C (PFAM: BTB, PF00651) and ecdysone-induced protein 75, E75 (PFAM: Hormone_recep and zf-C4, PF00104 and PF00105) (Zhang et al. 2018, Wu et al. 2021). The EFSB genome contains a single gene containing the canonical zinc finger domain (PFAM: zf-C2H2, PF00096) characteristic of Kr-h1. A single gene in the EFSB genome was annotated to be the E75 protein containing the Hormone_recep and zf-C4 PFAM domains, which was predicted to be spliced into 3 alternative transcripts. Similarly, the EFSB contains a single gene for Br-C having the characteristic BTB/POZ (PFAM: BTB, PF00651) and zinc finger (PFAM: zf-C2H2_6, PF13912) domains. The receptor for 20-E on the other hand is a heterodimer consisting of the ecdysone receptor (EcR) and the ultraspiracle protein (usp), the insect homolog of the mammalian retinoid X receptor (Iino et al. 2023). The EFSB contains a single gene having both the conserved DNA- (CDD: cd07161, NR_DBD_EcR) and ligand-binding (CDD: cd06938, NR_LBD_EcR) domains of the ecdysone receptor. However, the EFSB genome contains no clear annotation for the usp. There is a retinoid X receptor alpha gene having the Hormone_recep PFAM domain that may be the putative EFSB homologue of usp. More genes involved in hormone biosynthesis and signaling may be recovered upon improvement of the assembly and re-annotation. Nevertheless, analysis of the protein annotation files revealed the mostly complete set of core enzymes expected for chemosensation, detoxification, and biosynthesis of insect-specific hormones complemented by the expected genetic machinery for hormone signal transduction.

### Genetic Variations in the EFSB Genome

Genetic variations in the form of SNPs and Indels were determined using GATK. Recalibration of base quality scores is recommended by the GATK practices. Base score accuracies after recalibration show higher scores compared to before recalibration in both cycle and context covariates (Supplementary Fig. S6), indicating successful recalibration. There are some instances of the context covariate declining for specific contexts, but this is expected since there are cases when base calls are overestimated depending on the context of the previous nucleotide read.

**Fig. 4.**
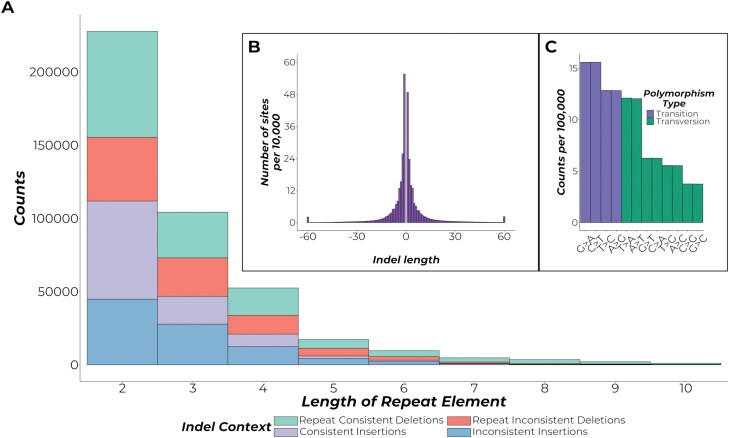
Summary of the obtained SNPs and Indels for the *Leucinodes orbonalis* EFSB genome. A). Summary of indels according to context. The plot shows the distribution of the obtained indels in terms of repeat length. The color shows the context of the indel changes whether the deletion or insertion is consistent with the repeat element associated with it. B) Distribution of indels according to the length of the indel. The *y*-axis shows the number of sites per 10,000 observations C) Summary of the SNPs according to type. The plot shows the abundance of each nucleotide change colorcoded according to the general polymorphism type, either transition or transversion. The y-axis shows the SNP count per 100,000 observations.

After variant calling, a total of 14,135,003 variants split into 11,103,848 SNPs and 3,031,155 Indels were detected. For the Indels, short repeats were more common than longer ones, showing an inversely proportional relationship between repeat length and abundance (Fig. 4A). Such an observation is corroborated in Fig. 4B, where the distribution of the indels according to length shows that most of the indels found are short variants. For the SNPs, it can be seen that transition polymorphisms are more common compared to transversions (Fig. 4C). The more common transition changes compared to transversions are known as the transition bias (Stoltzfus and Norris 2016). Overall, the filtered set of 14,135,003 genetic variants in the Philippine EFSB can be utilized to determine which genes are under positive or negative selection, and differentiate different subpopulations of the EFSB. Taken together, the genome assembly, annotation, and genetic variations obtained in this study can contribute to the scarce genetic resources of the EFSB, and aid in future research on the biology of the EFSB. Validation of the sequences and proteins obtained in this study could be utilized in developing complementary strategies for EFSB pest management.

## Supplementary Material

ieaf066_suppl_Supplementary_Figures_S1-S6

ieaf066_suppl_Supplementary_File

## Data Availability

The genomic sequencing reads utilized in this study have been deposited to the Sequencing Read Archive (SRA) with the following accessions numbers: SRR32012941 to SRR32012948 while the RNA-sequencing reads have been deposited to the SRA under the following accession numbers: SRR16214354 to SRR16214382. The SRA accessions for the genomic sequences are under BioProject PRJNA1211110 while the SRA accessions for the RNA sequences are under BioProject PRJNA768936. The combined assembly for the EFSB has been deposited to the NCBI Genomes database under accession number JBLEQA000000000. The whole genome annotation, additional supplementary information and other intermediate files were also uploaded to Zenodo and is available publicly at https://doi.org/10.5281/zenodo.15336556.
