# Supplementary material for: Nuclear genome assembly of Leucinodes orbonalis (Lepidoptera: Crambidae) collected from the Philippines
Source: J Insect Sci. 2025 Jun 28;25(3):24. doi: 10.1093/jisesa/ieaf066 (PMC12205365; doi:10.1093/jisesa/ieaf066)
Supplement: ieaf066_suppl_Supplementary_Figures_S1-S6 [file ieaf066_suppl_supplementary_figures_s1-s6.docx]

**Supplementary Information**


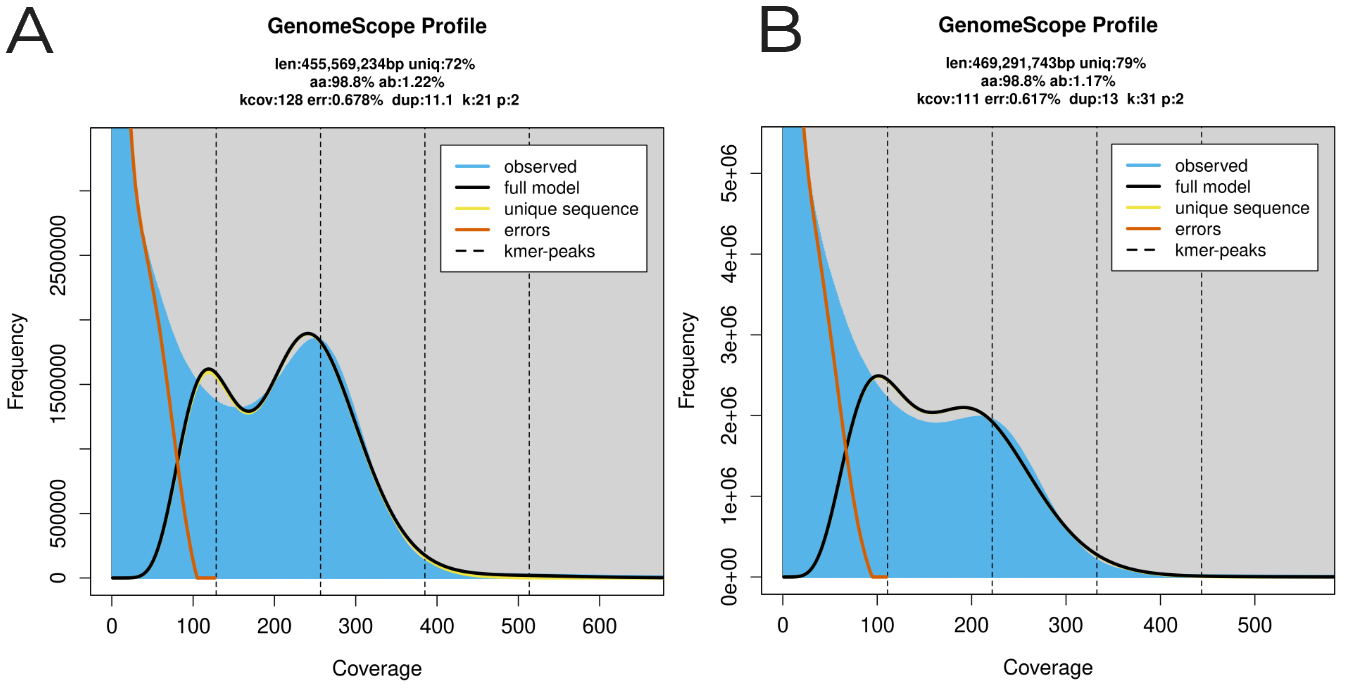


**Supplementary Fig. S1. K-mer based estimation of the *Leucinodes orbonalis*, eggplant fruit and shoot borer (EFSB) genome size using GenomeScope2.0.** The EFSB sequences were counted using Jellyfish using 21- and 31-mers, and the corresponding histograms were inputted into GenomeScope2.0 to estimate the genome size. The estimation using 21-mers (A) and 31-mers (B) revealed that the genome size (len) of the EFSB is around 455-469 Mb. The other parameters indicate the estimated percentage of the genome that is unique (uniq), estimated heterozygosity (ab), estimated mean k-mer coverage for the heterozygous regions (kcov), estimated error rate (err), and ploidy (p).

Supplementary Fig. S1 alt text. GenomeScope plot showing the estimated genome length for *Leucinodes orbonalis* using different k-mer lengths.


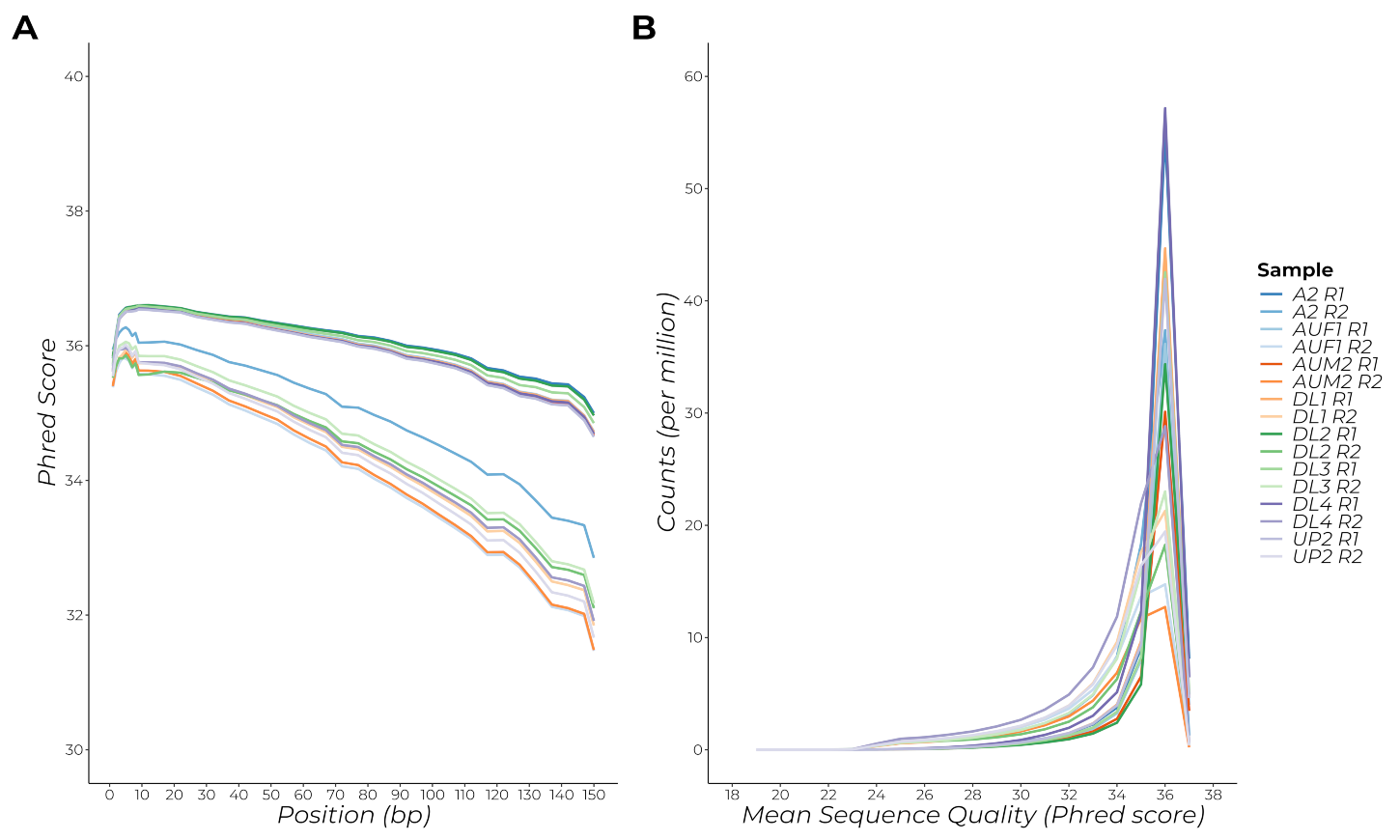


**Supplementary Fig. S2. Summary of read quality post-filtering of all sequencing libraries.** Qualities for the *Leucinodes orbonalis* sequencing reads after fastp filtering were assessed using fastqc and summarized with multiqc. The plot in **(A)** show the mean quality for each position in a read across all reads for each library while the plot in **(B)** show the mean quality of each read across all libraries. The legend indicates the libraries for each sample, which are deposited under NCBI BioSample as follows: A2, SAMN46265538; AUF1, SAMN46265539; AUM2, SAMN46265540; DL1, SAMN46265541; DL2, SAMN46265542; DL3, SAMN46265543; DL4, SAMN46265544; and UP2 SAMN46265545. Forward or reverse reads for each library are indicated by R1 or R2 respectively. Both plots show that the sequencing reads have high quality after filtering.

Supplementary Fig. S2 alt text. Line graph summarizing the base qualities of all sequencing reads used for assembling the *Leucinodes orbonalis* genome.


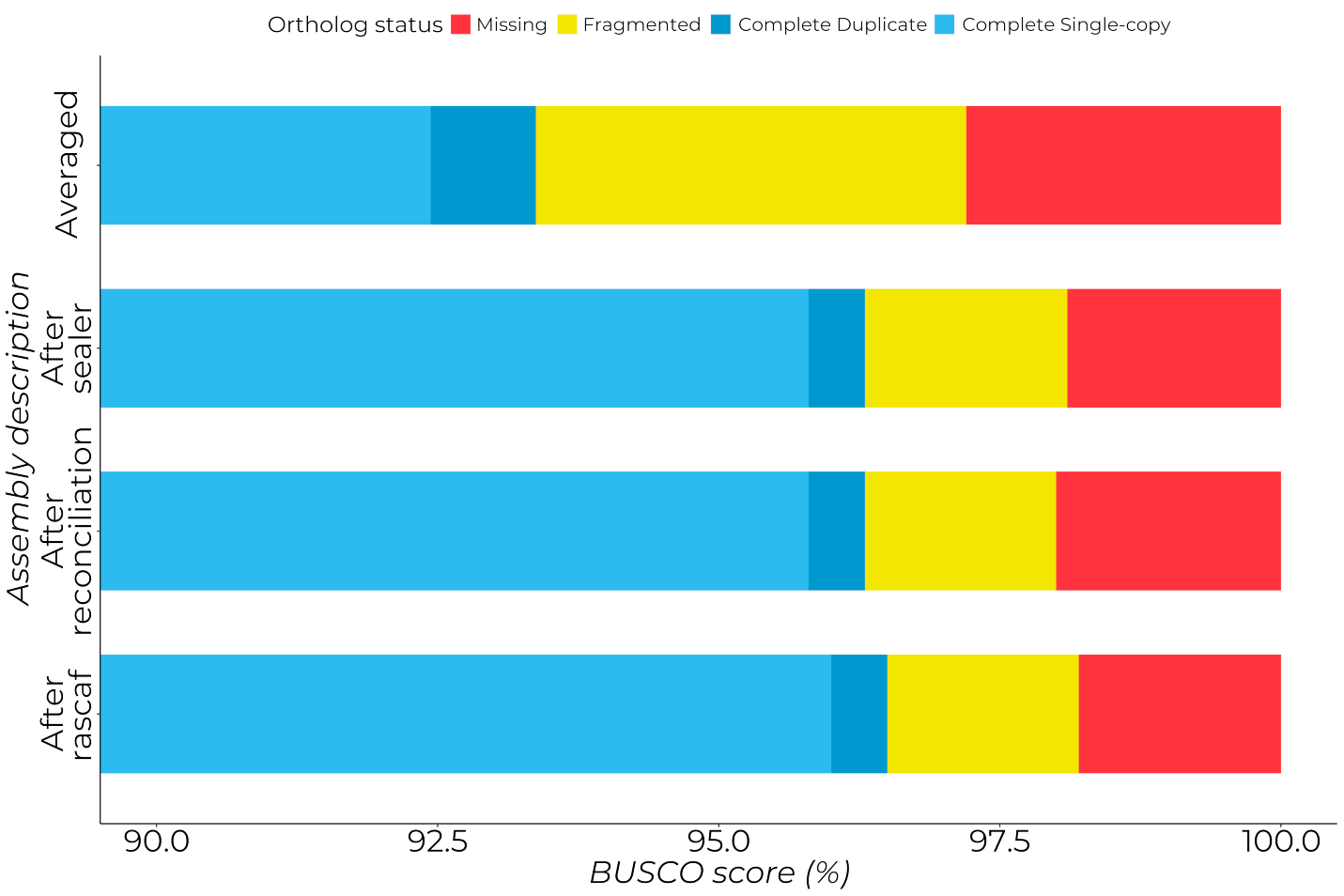


**Supplementary Fig. S3. BUSCO score comparison after each polishing step.** The averaged section indicates the mean BUSCO score across the individual 8 libraries, while the others indicate the assembly immediately after a polishing step. The colors depict the standard BUSCO color scheme of blue indicating complete BUSCOs while the yellow and red indicate fragmented and missing BUSCOs respectively.

Supplemenary Fig. S3 alt text. Bar graph comparison of quality checks after each major step during the genome assembly process.


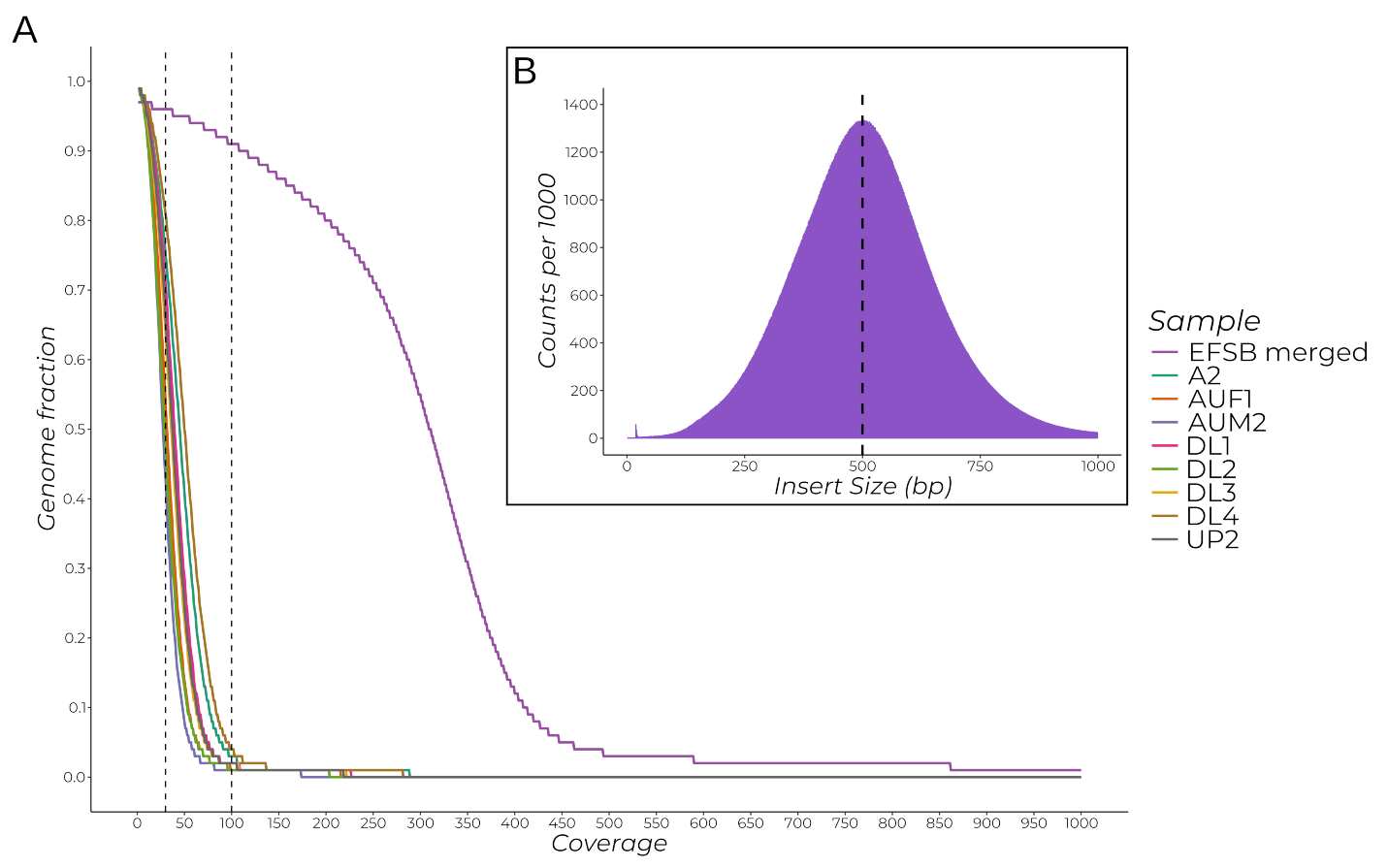


**Supplementary Fig. S4. Cumulative coverage distribution and insert size estimation of the merged and polished assembly. A).** Cumulative coverage distribution assessed by mosdepth showing the genome fraction per coverage value. The coverage of the merged and polished *Leucinodes orbonalis* assembly after rascaf was compared against the coverage of each individual assembly prior to merging. The vertical dashed lines indicate 30X and 100X coverage, showing that 96% and 91% of the merged assembly was covered at least 30 and 100 times respectively. **B).** Insert size distribution of the aligned reads assessed by picard tools. The mean insert size is 500 bp, close to the expected insert size produced during library preparation.

Supplementary Fig. S4 alt text. Chart summarizing the overall coverage and insert size distribution of the *Leucinodes orbonalis* genome.


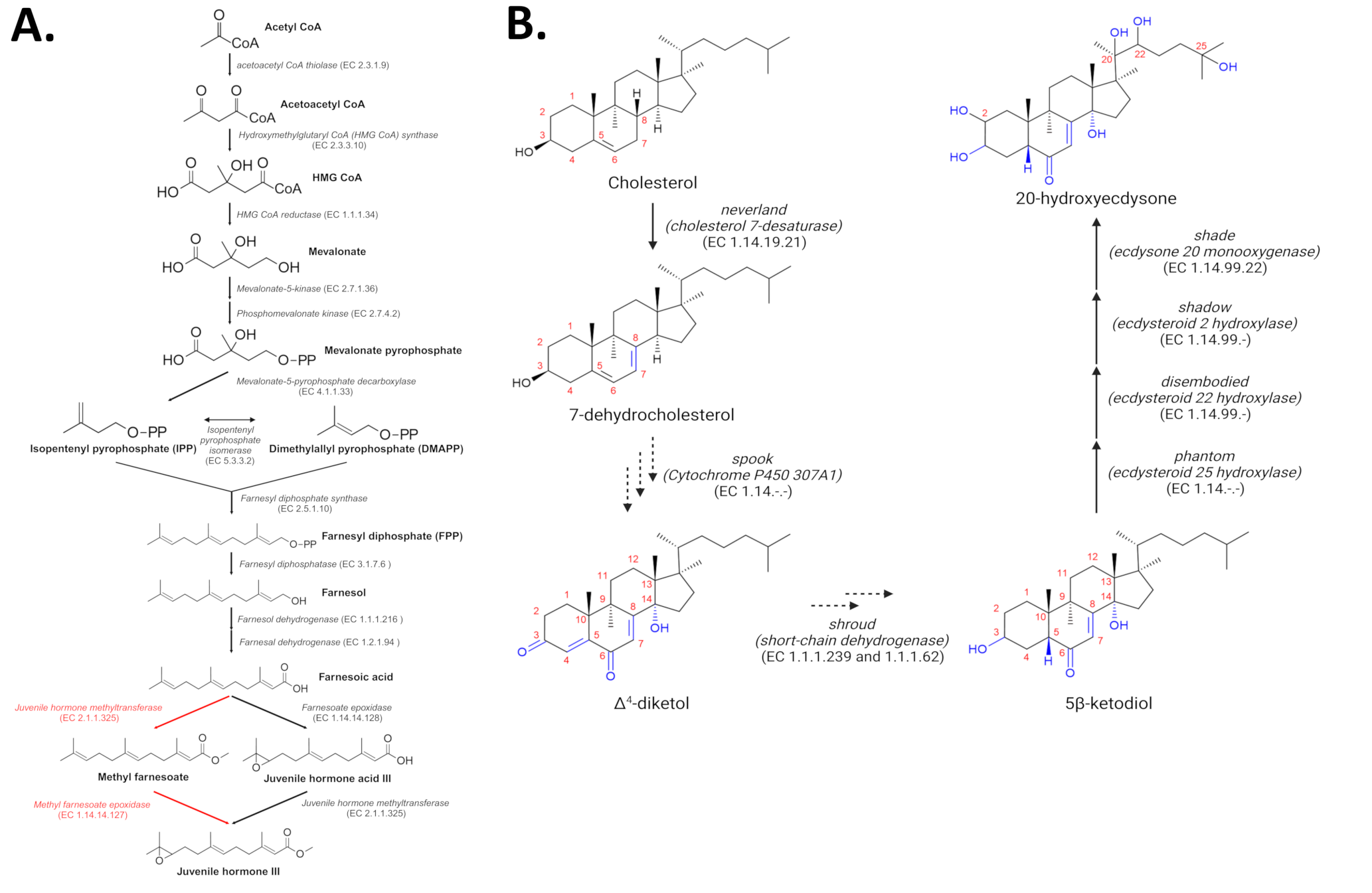


**Supplementary Fig. S5. Summary of insect hormone biosynthesis pathways. A).** Juvenile hormone (JH) biosynthesis. JH synthesis proceeds from the conserved mevalonate pathway that converts acetyl CoA into the isoprenoid building blocks IPP and DMAPP. Farnesyl diphosphate (FPP) is a key biosynthetic intermediate that can be shuttled to other biosynthetic pathways. For JH synthesis, FPP is successively converted to key intermediates through a variety of reactions culminating in farnesoic acid, which then gets epoxidated and methylated to form JH III. The reaction schemes in the black arrows are the putative biosynthetic steps in *Leucinodes orbonalis*, while the schemes in red arrows are the reactions occurring in non-Lepidopteran orders for comparison. **B).** Ecdysteroid biosynthesis (20-E). 20-E synthesis starts from dietary cholesterol, which is converted to 20-E through successive oxidation and hydroxylation reactions. Similarly for JH synthesis, the black arrows are the putative biosynthetic steps in *Leucinodes orbonalis*, while the dashed arrows are the Black box reactions described by Niwa and Niwa (2014). Important carbons are numbered in red for the cholesterol skeleton, and the successive changes to the cholesterol during 20-E synthesis are shown in blue. The molecule and reaction intermediate structures were drawn using ACD/Labs ChemSketch (ChemSketch 2024.1.0) while the whole reaction scheme was created using BioRender.

Supplementary Fig. S5 alt text. Reaction schemes for the key enzymes annotated in the assembled *Leucinodes orbonalis* genome involved in the biosynthesis of the major insect hormones.


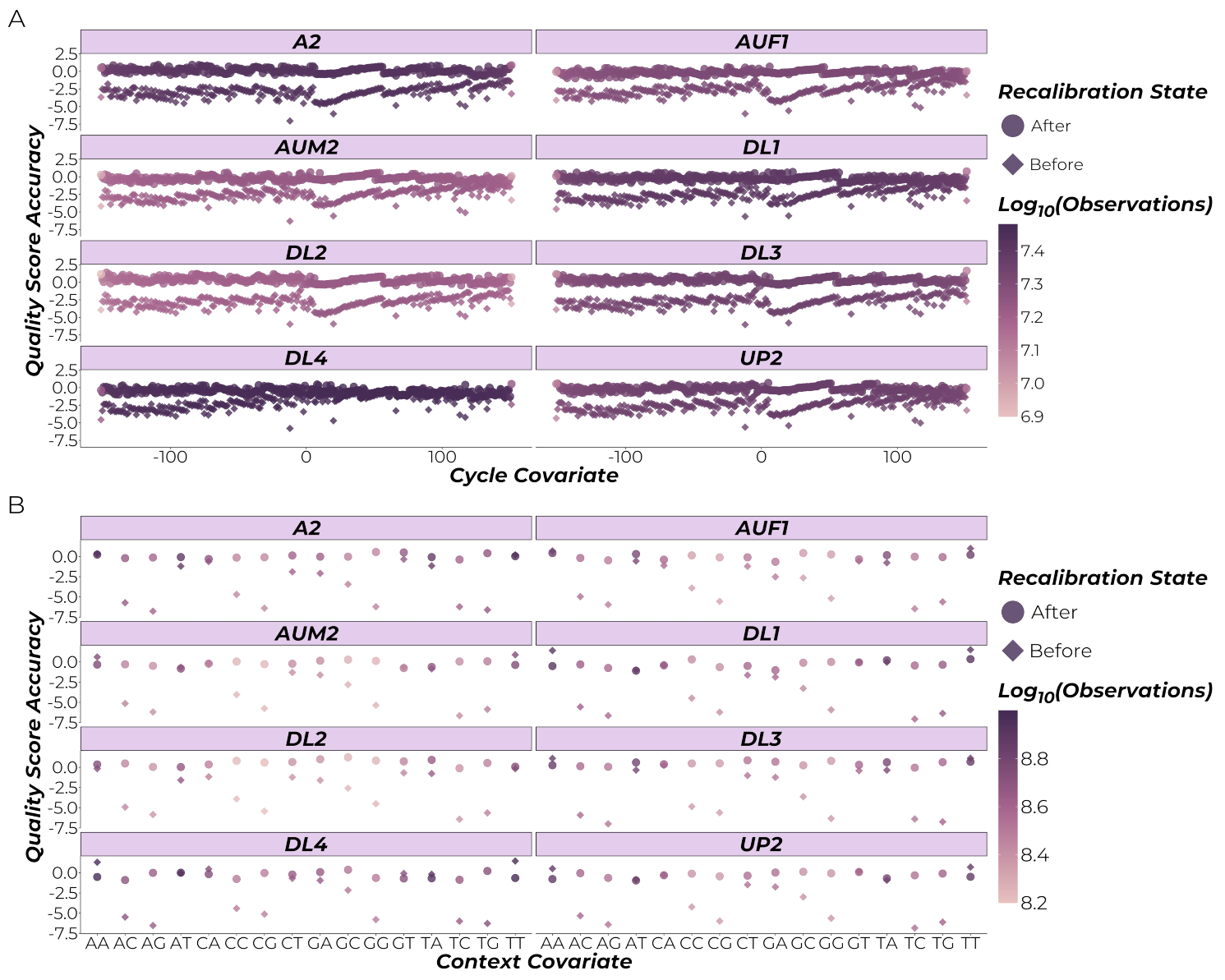


**Supplementary Fig. S6. Comparison of pre- and post-recalibration base quality scores with GATK Base Quality Score Recalibration (BQSR). A).** Quality Score Accuracy of the *Leucinodes orbonalis* sequencing reads based on cycle covariate for each library. The shape of the points represents before or after recalibration with BQSR, while the color represents the logarithm of the number of observations. **B).** Quality Score Accuracy of the *Leucinodes orbonalis* sequencing reads based on context covariate for each library. The shape and color of the points are the same as with **A).**

Supplementary Fig. S6 alt text. Comparison between pre- and post-base quality recalibration during the genetic variation profiling.
